# Supplementary material for: What Psychosocial and Physical Characteristics Differentiate Office Workers Who Develop Standing-Induced Low Back Pain? A Cross-Sectional Study
Source: Int J Environ Res Public Health. 2020 Sep 28;17(19):7104. doi: 10.3390/ijerph17197104 (PMC7579504; doi:10.3390/ijerph17197104)
Supplement: Supplementary file 1 [file ijerph-17-07104-s001.pdf]

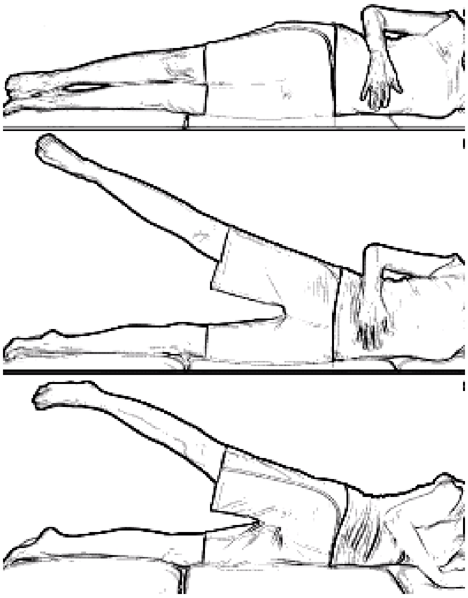

A. Active Hip Abduction (AHAbd) test

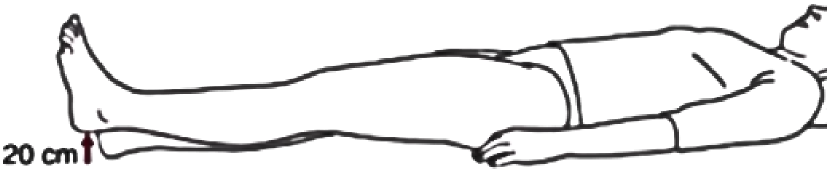

B. Active Straight Leg Raise (ASLR) test

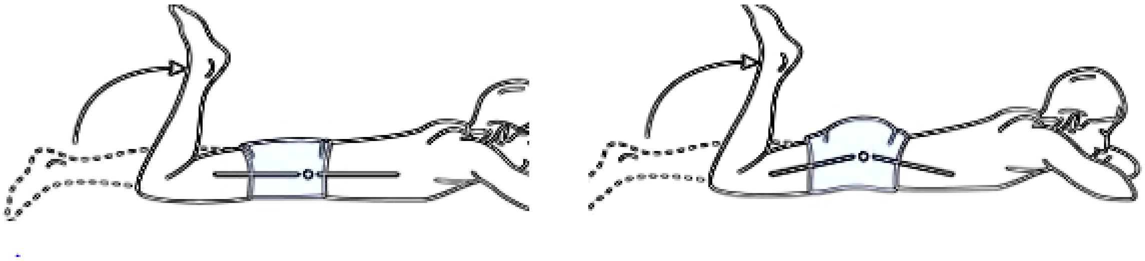

C. Prone Knee Flexion (PKF) test: correct (on the left), not correct (on the right)

Figure S1. Motor Control Test. (A) AHAbd test, (B) ASLR test, (C) PKF test

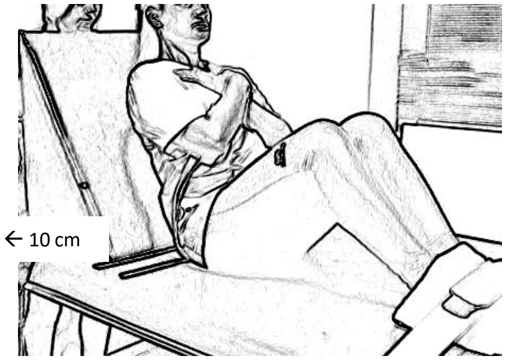

A. Abdominal endurance test

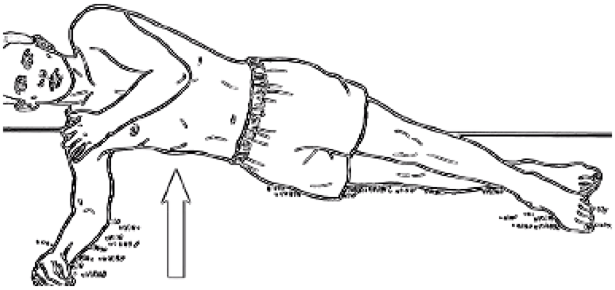

B. Side bridge test

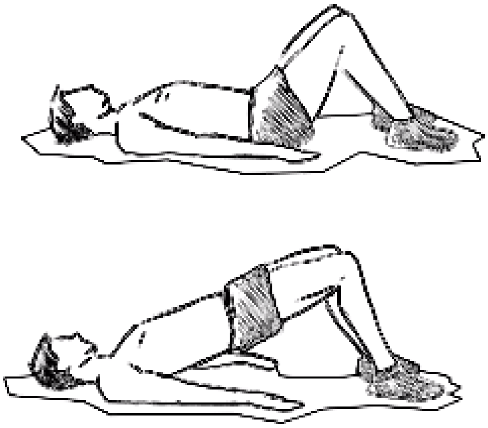

C. Supine bridge test

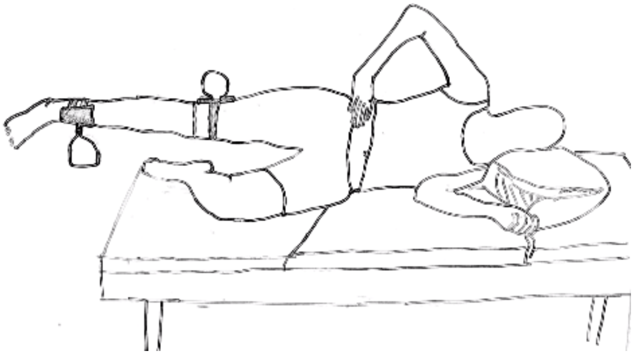

D. Isometric hip abduction test

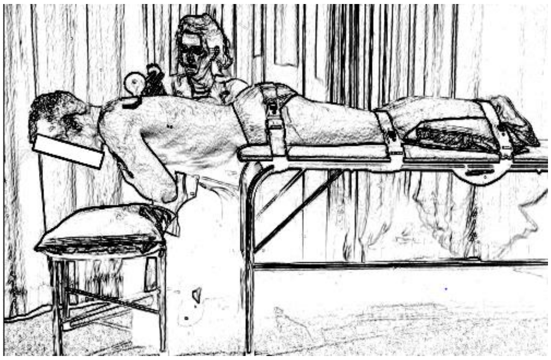

E. Biering-Sorensen test

Figure S2. Test of muscle endurance. (A) Abdominal, (B) Side bridge, (C) Supine Bridge, (D) Isometric hip abduction, (E) Biering- Sorensen test
